# Increased Risk of Renal Malignancy in Patients with Moderate to Severe Atopic Dermatitis

**DOI:** 10.3390/cancers15205007

**Published:** 2023-10-16

**Authors:** Jongwook Oh, Hyun Ju Oh, Kyung-Do Han, Heon Yung Gee, Ji Hyun Lee

**Affiliations:** 1 Department of Pharmacology, Yonsei University College of Medicine, Seoul 03722, Republic of Korea; 2 Department of Medicine, Physician-Scientist Program, Yonsei University Graduate School of Medicine, Seoul 03722, Republic of Korea; 3 Department of Dermatology, Seoul St. Mary’s Hospital, College of Medicine, The Catholic University of Korea, Seoul 06591, Republic of Korea; 4 Department of Statistics and Actuarial Science, Soongsil University, Seoul 06978, Republic of Korea; 5 Graduate School of Medical Science, Brain Korea 21 Project, Yonsei University College of Medicine, Seoul 03722, Republic of Korea

**Keywords:** atopic dermatitis, renal malignancy, systemic steroid, Korean, cohort study

## Abstract

**Simple Summary:**

Research suggests that a skin condition called atopic dermatitis (AD) and cancer, specifically kidney cancer, might be connected. However, we do not have enough evidence to confirm this link. This study aimed to determine whether the risk is real and whether the severity of AD influences this association. We used a big database of health information from Korea for the investigation and found that people with moderate to severe AD have a higher-than-average chance of getting kidney cancer. This discovery could help doctors better understand these conditions and might lead to more frequent check-ups for kidney cancer in people with AD, especially those with severe disease.

**Abstract:**

Background: Evidence for an association between atopic dermatitis (AD) and cancer is still insufficient. In particular, the association between the risk of renal malignancy and the severity of AD has not been thoroughly investigated. Objective: To investigate the risk of renal malignancy and determine the association between AD severity and cancer risk using data from the Korean National Health Insurance Service (KNHIS) database. Methods: We performed a population-based cohort study using the National Health Claims database from the NHIS in Korea. Results: We found a statistically significant association between AD and overall malignancy (for mild AD, hazard ratio (HR): 1.061, 95% confidence interval (CI): 1.006–1.118; for moderate to severe AD, HR: 1.061, 95% CI: 1.014–1.11) compared with the no AD group. The moderate to severe AD group showed a significantly increased risk for renal malignancy (adjusted HR: 1.533, 95% CI: 1.209–1.944) compared with the no AD group. Limitations: Patient inclusion is solely based on diagnostic codes. We had no data about drug use, genetic factors, or other medical history that could affect the cancer risk. Conclusion: In our large population-based cohort study, moderate to severe AD was associated with increased risk of renal malignancy. Regular check-ups for renal malignancy are recommended in this population.

## 1. Introduction

Atopic dermatitis (AD) is the most common chronic inflammatory skin disorder, with a lifetime prevalence of 15–20% in developed countries [1]. AD affects up to 10% of adults and 20% of children [2]. The central pathogenesis of AD is impairment of the skin barrier function and aberrant immune function toward the T helper 2 cell-mediated immune response [3]. AD usually evolves as a chronic disease, and its course can be continuous for long periods or of a relapsing nature with repeated flare-ups triggered by viral, bacterial, or fungal infections; irritants; psychosocial factors; etc. [4]. The diagnosis is made clinically through history taking and physical examination.

Globally, cancer is the leading cause of death, and its socio-economic effects are enormous [5,6]. Given AD’s chronicity, it is important to study the association between AD and cancer risk. Evidence is accumulating that AD could influence cancer risk. Impairment of the skin barrier function could facilitate the entry of potential carcinogenic agents or viruses, and immune dysregulation could alter immune surveillance of cancer. Many observational epidemiologic studies have yielded extensive data about the association between AD and cancer [7,8,9]. Although data on the cancer risk of AD patients are conflicting, a recent systematic review and meta-analysis highlighted the evidence of a cancer risk specific to AD patients [10,11]. However, renal malignancy has not been investigated thoroughly. The previous reviews showed that AD has a tendency to increase the risk of renal malignancy. However, the available review data were derived from two studies, which were conducted for short periods of time in small cohorts.

Treatment goals in AD are to reduce symptoms such as pruritus and rash and establish persistent disease control. Treatment selection is based largely on disease severity. In mild-to-moderate AD, topical corticosteroids or calcineurin inhibitors are appropriate. Systemic therapy, such as cyclosporin, methotrexate, steroid, or biologics targeting specific pathogenesis factors, is recommended for persistent, moderate-to-severe AD that is not controlled by topical agents. Recently, probiotic therapy has also emerged as a preventive treatment [12,13]. Systemic corticosteroids are commonly used as a first-line systemic treatment for AD, typically in short courses to suppress disease activity and manage acute flares [14]. Many reviews of the safety and efficacy of systemic agents have strongly recommended the short-term use of systemic agents [14,15,16]. Although a malignancy risk is mentioned, the observation period for these safety data is only 52 weeks, so the long-term safety of systemic agents for AD is not fully understood. Also, given the need for long-term AD management, an investigation of the association between AD and overall malignancy has important clinical implications.

Therefore, our primary objective in this study was to determine the association between AD and the risk of renal malignancy. In addition, we conducted stratified analyses based on AD severity. The severity analysis could demonstrate a valuable association between AD and the risk of malignancy. Our secondary objective was to evaluate the risk of renal malignancy according to treatment agents for AD in data from the Korean National Health Insurance Service (KNHIS) database.

## 2. Materials and Methods

### 2.1. Study Design and Database

We performed a population-based cohort study using the KNHIS National Health Claims database. KNHIS is managed by the Korean government and covers almost 100% of the Korean population. All medical diagnoses and prescription records of Koreans are stored in KNHIS databases. The KNHIS-National Health Screening Cohort (KNHIS-HEALS) database comprises a complete set of health data; a detailed profile of the KNHIS-HEALS cohort is provided elsewhere [17]. Screening programs are conducted every 2 years for Koreans older than 20 years. The KNHIS-HEALS database is a healthcare service claims database that contains inpatient and outpatient medical records and health screening data.

To evaluate the risk of renal malignancy according to AD treatment, the following information was collected from the database: prescribed topical agents, prescribed oral medications (corticosteroid, azathioprine, cyclosporine, methotrexate, and mycophenolate mofetil), and doses during the study period.

This study was approved by the institutional review board of the Catholic University of Korea (approval no. KC21ZISI0966).

### 2.2. Study Population

**Figure 1:**
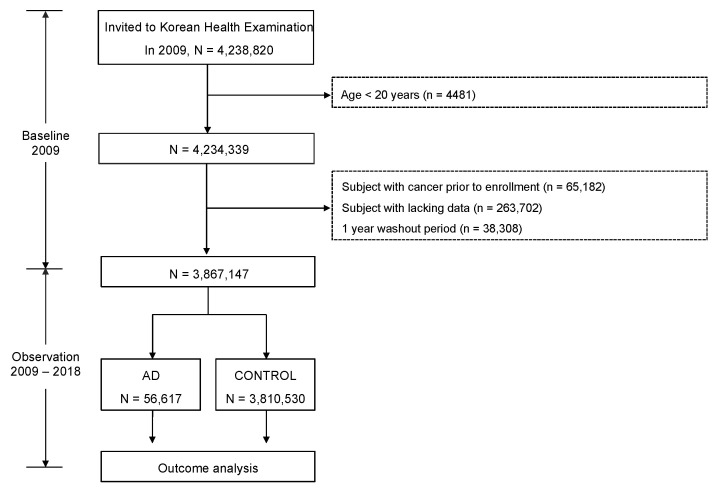
Flow chart of study population enrollment.

In the baseline period (2009), we extracted a sample cohort of 4,238,820 adults from KNHIS. To clarify the correlation between AD and cancer risk, 65,182 subjects diagnosed with cancer before enrollment were excluded. Also, 263,702 subjects lacking data were excluded. In addition, 38,308 subjects with previously diagnosed AD were excluded. Thus, the study began after a one-year washout period from 2008 to 2009 to reduce the confounding effect of previously diagnosed AD. Therefore, 3,867,147 subjects were recruited (Figure 1). The median follow-up duration was 8.29 years.

### 2.3. Inclusion Criteria for AD

A diagnosis of AD was confirmed using ICD-10 codes (L20) in the medical records of outpatients and inpatients. We enrolled AD patients who had at least three hospital visits for AD in one year. Based on treatment, we categorized participants as no AD, mild AD, and moderate-to-severe AD. Patients with AD who received at least one systemic treatment (cyclosporin, methotrexate, retinoids, azathioprine, phototherapy, or biologic agents) were considered to have moderate to severe AD. Patients with AD who did not receive any systemic treatment were deemed to have mild AD.

### 2.4. Data Collection

The outcome of interest was the development of malignancy, defined as a record of a relevant ICD-10 code. In Korea, patients who are diagnosed with cancer are enrolled in a cancer registry to receive governmental financial support based on their diagnosis. Therefore, cancer diagnoses based on ICD-10 codes are highly reliable.

Overall malignancy comprised the following ICD codes: stomach (C16), colorectal (C18–20), liver (C22), pancreatic (C25), lung (C33–34), thyroid (C73), lymphoma (C82–86), oral (C00–C14), esophagus (C15), biliary (C23–24), laryngeal (C32), renal (C64), bladder (C67), nerves (C70–72), multiple myeloma (C90), leukemia (C91–95), skin (C43), breast (C50), cervical (C53), corpus (C54–55), ovarian (C56), prostate (C61), and testicular (C62). Among skin malignancies, only melanoma was included in our analyses. We performed subgroup analyses by AD severity to investigate the risk of malignancy.

### 2.5. Statistical Analysis

The study population was classified based on AD severity. For baseline comparisons, Student’s *t*-test or the Mann–Whitney U test was performed for continuous variables, and the chi-square test was used for categorical variables. Each malignancy’s incidence rate was calculated by dividing the total number of incident cases by the entire follow-up in person-years. We performed multivariate Cox proportional hazard regression analyses to evaluate the associations between AD severity and the risk of malignancy. The multivariate model controlled for baseline age, sex, body mass index (BMI), smoking, drinking, physical activity, hypertension, DM, and dyslipidemia. All these variables are based on baseline time-point.

Age was divided into three groups (20–39, 40–64, 65≥). We also performed subgroup analyses by treatment modality. The topical group used only topical agents. The systemic steroid group received systemic steroid treatment as the only oral medication. The other systemic agent group received other systemic agents (cyclosporin, azathioprine, methotrexate, or mycophenolate mofetil). All statistical analyses were performed using SAS software (version 9.4, SAS Institute, Cary, NC, USA). *p* Values < 0.05 were considered statistically significant.

## 3. Results

### 3.1. Characteristics of the Study Population by AD Severity

Among the 3,867,147 healthy individuals recruited, 22,430 developed mild AD, and 34,187 developed moderate to severe AD. Generally, baseline demographics did not differ significantly among the three groups. The proportion of individuals younger than 40 was higher in the moderate to severe AD group (35.35%) than in the mild AD group (30.38%) and no AD group (31.58%). The proportion of participants in the old age group (>65) was highest in the mild AD group. The proportion of males was highest in the no AD group, and the proportion of females was higher in the mild and moderate to severe AD groups. Non-smoking was highest in the moderate to severe group, and current smoking was highest in the no AD group. The proportion of patients from the ex-smoking group in each AD group was similar. Heavy drinking had the highest ratio in the no AD group, and non-drinking had the highest ratio in the mild AD group. The rate of regular exercise was similar in the three groups. The ratio of DM, hypertension, dyslipidemia, and chronic kidney disease was highest in the mild AD group without a significant difference between the no AD group and the moderate to severe AD group. Also, no differences among the groups were found in the BMI, waist circumference, blood glucose level, or cholesterol. In the moderate to severe AD group, most patients received systemic steroids (99.42%). The next most used drug was cyclosporine (1.26%), which was followed by methotrexate (0.19%), azathioprine (0.08%), and mycophenolate mofetil (0.06%) (Table 1).

### 3.2. Incidence Rate and the Risk of Malignancy in the Study Population by AD Severity

After about eight years of follow-up, the AD group showed a higher occurrence of overall malignancy. The incidence ratio of overall malignancy was 6.92 in the moderate to severe AD group, 7.7 in the mild AD group, and 6.56 in the no AD group (Table 2). The moderate to severe AD group showed a significantly increased risk of overall malignancy (hazard ratio (HR): 1.055, 95% confidence interval (CI): 1.008–1.104) compared with the no AD group. First, adjusting for age and sex, the moderate to severe AD group showed a 5.7% higher risk for overall malignancy (adjusted HR: 1.057, 95% CI: 1.01–1.106) than the reference group. After adjusting for age, sex, DM, hypertension, dyslipidemia, smoking, alcohol intake, exercise status, and BMI, patients in the moderate to severe AD group had a 6.1% higher risk of overall malignancy (adjusted HR: 1.061, 95% CI: 1.014–1.11) than the reference group.

During follow-up, 5157 events of renal malignancy occurred in the 3,810,530 no AD participants, 30 events in the mild AD group, and 69 events in the severe AD group, for ratios of 0.16447, 0.16287, and 0.24604, respectively. The moderate to severe AD group showed a significantly increased risk of renal malignancy (HR: 1.496, 95% CI: 1.18–1.897) compared with the no AD group. After adjusting for age, sex, DM, hypertension, dyslipidemia, smoking, alcohol intake, exercise status, and BMI, the moderate to severe AD group still had a significantly increased risk of renal malignancy (adjusted HR: 1.533, 95% CI: 1.209–1.944) compared with the no AD group.

### 3.3. Incidence Rate and Risk of Malignancy in the Study Population by Treatment Modality

We also investigated the effect of treatment modality on renal malignancy (Table 3). During follow-up, 5157 events of renal malignancy occurred in the 3,810,530 no AD participants, 24 events in the 19,892 participants who received only topical AD treatments, 68 events in the 33,988 participants who received systemic steroid AD treatment, and seven events in the 2737 participants who received other systemic agents for AD, for ratios of 0.16447, 0.14684, 0.24389, and 0.3128, respectively. Topical agents did not increase the risk of developing renal malignancy compared with the reference group (HR: 0.893, 95% CI: 0.567–1.265). After adjusting for confounding factors, topical agents showed the same tendency as the reference group (adjusted HR: 0.828, 95% CI: 0.555–1.237). The systemic steroid use group had a significantly increased risk of renal malignancy (HR: 1.483, 95% CI: 1.168–1.884) compared with the reference group. After adjusting for several confounding factors, the positive association between systemic steroids and renal tumors was maintained (adjusted HR: 1.519, 95% CI: 1.196–1.93). The other systemic agents group also showed an increased risk, but the increase was not statistically significant (HR: 1.903, 95% CI: 0.907–3.993). After adjusting for confounding factors, the other systemic agents’ group did not have a statistically significant risk of renal cancer (adjusted HR: 1.476, 95% CI: 0.703–3.098).

## 4. Discussion

AD usually occurs in early childhood and is increasingly recognized as a disease that often persists into adulthood [18]. AD can detrimentally influence quality of life and imposes social and financial burdens [19]. Due to itching, scratching, effects on sleep, and treatment, AD can profoundly influence the quality of life in childhood [20]. The skin, as an important organ of external appearance, contributes to social standing. One survey also showed that quality of life is affected in adult AD [21]. AD has an economic effect on patients and their families. The total annual burden of AD was USD 4.228 billion in 2004, compared with USD 3.658 billion for psoriasis, including both direct and indirect costs (including lost productivity of patients and caregivers and lost productivity due to early mortality) [22]. In that study, AD had higher indirect costs than psoriasis. Globally, cancer is the leading cause of death, and its socio-economic effects are also enormous [5,6]. Thus, the extent of the burden imposed by AD is exacerbated through cancer generation, and it is greatly important to study the association between AD and cancer risk.

Our large, population-based cohort study showed that moderate to severe AD patients had a higher incidence rate of overall malignancy compared with healthy individuals after adjustment for known cancer risk factors such as smoking, excessive alcohol consumption, and obesity. Other large population-based cohort studies showed small evidence for an overall malignancy risk. An English cohort showed minimal increased risk for malignancy, and the increase reported for the Danish cohort was not statistically significant (adjusted HR: 1.04, 99% CI: 1.02–1.06 in England; adjusted HR: 1.05, 99% CI: 0.95–1.16 in Denmark). However, in the severity analysis, severe AD showed an increased risk for overall malignancy (adjusted HR: 1.16, 95% CI: 1.07–1.26). Despite conflicting data on the risk of cancer in patients with AD [23,24], the risk tends to increase as severity increases, suggesting that AD might affect the cancer risk.

The results of two large studies indicated that the risk differed by cancer type [10,11]. Wang’s systematic review reported a statistically significant association between AD and keratinocyte carcinoma (pooled SIR of five studies: 1.46, 95% CI: 1.20–1.77) [7,9,25,26,27]. Mansfield’s cohort study showed small increases in the nonmelanoma skin cancer risk (adjusted HR: 1.11, 99% CI: 1.06–1.15 in England; adjusted HR: 1.17; 99% CI: 0.99–1.38 in Denmark). Both studies reported no statistically significant association between AD and melanoma. Several factors could explain the conflicting associations between skin malignancy and AD. AD patients are likely to have more frequent skin exams than people without AD, which could lead to a decreased risk of melanoma due to better detection at an early stage or an increased rate of malignancy detection because of frequent skin exams. An impaired skin barrier could increase the keratinocyte damage caused by UV. Additional studies are needed to elucidate the association between AD and skin malignancy.

Wang’s study showed an increased risk of central nervous system (CNS) (pooled SIR of two studies: 1.81, 95% CI: 1.22–2.70), and pancreatic disease (SIR of one study: 1.90, 95% CI: 1.03–3.50) [7,9]. However, no statistically significant association was found between CNS disease and AD (adjusted HR: 0.99, 99% CI: 0.83–1.18 in England; adjusted HR: 0.78, 99% CI: 0.41–1.50 in Denmark) or pancreatic disease and AD (adjusted HR: 0.99, 99% CI: 0.86–1.14 in England; adjusted HR: 1.62, 99% CI: 0.85–3.09 in Denmark). No association with diseases of the pancreas or CNS was found as AD severity increased. Halling-Overgaard reported an inverse association between AD and brain cancer and no association with the pancreas [28]. For CNS and pancreatic malignancy, the evidence is insufficient to suggest a conclusion, so further studies are needed.

For lung malignancy, Wang’s study showed a statistically significant association in case-control studies (pooled odds ratio of four studies: 0.61, 95% CI: 0.45–0.82) [6,29,30]. Although a small increase in lung cancer risk was shown in Mansfield’s cohort study, the association disappeared after adjusting for confounding factors. Taken together, the evidence is insufficient, and we cannot reliably assess the risk of lung malignancy in AD patients.

Two previous papers showed that AD tended to increase the risk of renal malignancy, but the association was not statistically significant [7,9]. In Wang’s pooled meta-analysis results, the risk of kidney cancer was reported to be 1.86 [11]. The two previous studies used quite small populations and did not adjust for confounding factors such as smoking or hypertension, and the meta-analysis result was derived from those two studies. Our results show the same trend. The moderate to severe AD group showed about a 53% increase in risk compared with the general population (HR: 1.519, 95% CI: 1.196–1.93). Our large population-based study was adjusted for numerous potential confounders, especially known renal cancer risk factors such as BMI, smoking, and HTN [31]. In addition, we performed an analysis according to AD severity and found a valuable association.

Wang’s study reported no evidence of an association between AD and breast cancer. Likewise, Mansfield’s cohort study found no strong evidence for an association between AD and breast cancer (adjusted HR: 0.98, 99% CI: 0.92–1.03 in England; adjusted HR: 0.97, 99% CI: 0.76–1.24 in Denmark). No association was found in the analysis according to severity (adjusted HR: 1.02, 99% CI: 0.91–1.14).

Both studies reported no or insufficient evidence for an association between AD and other cancer types, including colorectal, head and neck, male genitourinary, and gastrointestinal malignancies.

An increased risk of lymphoma was seen among people with AD in England (adjusted HR: 1.19, 99% CI: 1.07–1.34 for non-Hodgkin lymphoma; adjusted HR: 1.48, 99% CI: 1.07–2.04 for Hodgkin lymphoma). In the severity analysis, the risk increased as the severity increased (non-Hodgkin lymphoma adjusted HR: 1.06, 99% CI: 0.90–1.25 for mild AD; NHL adjusted HR: 1.24, 99% CI: 1.04–1.48 for moderate AD; NHL adjusted HR: 2.08, 99% CI: 1.42–3.04 for severe AD).

Another meta-analysis [32] reported that the risk of lymphoma increased, with a relative risk of 1.43 (95% CI: 1.12–1.81) in cohort studies. Case-control studies showed no significant increase in the risk of lymphoma. In that review, AD severity was a significant risk factor for lymphoma. In three studies that evaluated the potential risk of lymphoma associated with topical corticosteroids (TCSs), the use of high potency TCSs significantly increased the risk of lymphoma, with an overall OR of 1.73 (95% CI: 1.52–1.97). However, that result is unclear because AD severity might have confounded the risk of lymphoma. Patients with severe AD are generally treated with highly potent TCSs. In Arellano’s case-control study, the increased risk of lymphoma associated with TCSs disappeared after adjusting for AD severity [33], which suggests that the association between high-potency steroid use and lymphoma is doubtful. Taken together, the correlation between AD and lymphoma seems reliable, but further research on the association between highly potent TCSs and the risk of lymphoma is needed. In addition, there is a difference in the results according to the type of lymphoma, such as non-Hodgkin lymphoma and Hodgkin lymphoma, so further study about this will be needed.

AD is a complex, multifactorial disease, and several hypotheses have been proposed to explain the association between AD and the development of malignancy. First, AD patients might show an increased detection of skin cancer because of frequent follow-up for their chronic skin disease. If AD is severe, patients have a high possibility of taking systemic agents that require regular blood tests to detect abnormalities. Nonetheless, the association might not be entirely due to ascertainment bias because AD could influence the risk of malignancy. Some reports have indicated that the use of topical tacrolimus, pimecrolimus, and topical corticosteroids could increase the risk of lymphoma and skin cancer [32,34]. Because patients with more severe AD are likely to use greater amounts of those drugs than those with less severe disease, it is possible that the drug usage might be the cause of the increased cancer risk.

Second, impairment of the skin barrier due to a loss of function of the filaggrin gene (FLG) might also contribute to the development of malignancy by increasing UV-induced damage to keratinocytes or exposure to other carcinogens. A case study showed that individuals with filaggrin mutation had an increased risk of squamous cell carcinoma (SCC) [35], indicating a potential association between the development of SCC and filaggrin mutation. The skin barrier function is impaired in AD patients with FLG mutations, and FLG mutations are significantly associated with a higher median total epidermal water loss. One risk factor for SCC is human papilloma virus infection [36]. Epidermal barrier dysfunction leads to increased permeability of low-molecular-mass chemicals and susceptibility to infection [37,38,39]. Clinically, patients with AD often develop severe forms of viral infection, such as eczema herpeticum, which is seldom seen in healthy people [40]. Cohort studies also suggest that AD patients might be at risk of non-cutaneous infections [41].

Third, chronic inflammation might also explain the possible association between AD and cancer. One characteristic of AD is helper T cell subtype-2 hyperreactivity-driven skin inflammation. Chronic inflammatory status could negatively or protectively affect the cancer risk [42,43,44,45,46]. Tumor-infiltrating immune cells can either promote or inhibit cancer development, depending on the immune–cancer interaction [47,48]. By producing cytokines, chemokines, and other enzymes, tumor-infiltrating immune cells can advance tumor progression by promoting cell proliferation and inhibiting programmed cell death [49,50]. Pompei showed that patients with allergies had an increased response to cancer treatment and better cure rates than controls [46]. Conversely, chronic inflammation might lead to the development of keratinocyte cancer by increasing cell turnover. Chronic inflammation could contribute to cancer development and predispose individuals to carcinogenesis [51]. Inflammation due to infection is involved in the development of about 15% of human tumors [45]. An infection-induced inflammatory state is a risk factor for the development of several carcinomas [52]. The association between cancer and inflammation is thus reliable. Recently, ‘tumor-promoting inflammation’ has been included as a cancer hallmark [53].

We aimed to determine the association between AD treatment and the risk of renal malignancy. A subgroup analysis of AD therapeutic agents and the risk of renal malignancy showed that the risk of malignancy was higher in patients using systemic steroids than in others. In the group using only topical agents, no association with malignancy was found. A previous review of small studies investigating systemic steroids for AD made a brief mention of a malignancy risk [14]. It is significant that this large-scale study indicates that systemic steroid use in AD patients could increase the risk of renal malignancy. One cancer hallmark is its ability to avoid immune destruction, which would otherwise operate as a barrier to tumor formation and progression [53]. From that point of view, immune suppression by systemic steroids might also contribute to the malignancy risk. However, recent long-term follow-up studies of topical tacrolimus and corticosteroid treatment reported no evidence of an increased cancer risk in children with AD [54]. Thus, it is challenging to evaluate the exact effect of systemic steroids on the development of renal malignancy in AD patients. Some studies have shown that systemic glucocorticoid exposure was associated with non-Hodgkin lymphoma, bladder cancer, and SCC [44,55,56]. Conversely, no association was reported between systemic steroids and the risk of colorectal cancer [57]. Cancer-specific differences in immunosuppression are supposed, and previous papers have already shown different risks for each type of cancer. For renal malignancy, sophisticated studies on the effects of systemic steroids are needed.

Our study has certain strengths. We used a large, population-based, and highly representative database of more than 3 million subjects to analyze the risk of overall malignancy during 8 years of follow-up. In addition, we analyzed the severity of AD and performed a subgroup analysis by treatment to determine the association with renal malignancy. Few previous data on renal malignancy are available, and no previous studies have addressed the risk associated with treatment agents. Thus, our results offer a better understanding of the mechanisms underlying the possible association between AD and renal malignancy.

Nonetheless, this study also has several limitations, and the results should be interpreted carefully. First, the study design is retrospective. Because the study population size is large and the observation period is long, variables at baseline could be inaccurate. In other words, a limitation of our study is that we used only baseline values for time-varying factors. Also, our results establish only an association, not a cause–effect relationship between AD and malignancy. Second, the use of ICD-10 code-based data in KNHIS carries the possibility of disease misclassification. Third, we cannot exclude ascertainment bias; cancers might be diagnosed more frequently in individuals with severe AD because they receive more regular skin examinations and blood tests than others. Fourth, our dataset lacked information about genetic factors, medication doses and intervals, other drug history, and other medical history, which could influence the malignancy risk. However, we used a multivariable Cox proportional hazards model to adjust for correctable confounding factors.

## 5. Conclusions

In conclusion, our large, population-based cohort study revealed that AD is associated with an increased risk of overall malignancy in the Korean population. Our data highlight that patients with moderate to severe AD are more vulnerable to renal malignancy than others. Also, systemic corticosteroid use might be a potential risk factor for renal malignancy in patients with AD. Regular check-ups for renal malignancy are recommended for patients with severe AD. It is challenging to draw firm conclusions from the epidemiological data. Therefore, further studies with clearly defined criteria for AD diagnosis and the ability to adjust for other key confounding factors are required to understand the possible association between AD and cancer risk.

## Figures and Tables

**Table 1:** Baseline demographics of the study population according to AD severity.

Characteristics		AD Severity			
		No AD	Mild	Moderate to Severe	*p*-Value
		3,810,530	22,430	34,187	
Age, years		46.96 ± 13.96	48.47 ± 15.21	46.46 ± 15.16	<0.0001
Age, years (group)	*n*, (%)				<0.0001
<40		1,203,545 (31.58)	6815 (30.38)	12,085 (35.35)	
40–64		1,826,410 (47.93)	9556 (42.6)	14,388 (42.09)	
≥65		780,575 (20.48)	6059 (27.01)	7714 (22.56)	
Sex	*n*, (%)				<0.0001
Male		2,092,135 (54.9)	10,127 (45.15)	15,765 (46.11)	
Female		1,718,395 (45.1)	12,303 (54.85)	18,422 (53.89)	
Smoking	*n*, (%)				<0.0001
None		2,258,682 (59.27)	15,013 (66.93)	22,143 (64.77)	
Ex		543,602 (14.27)	3240 (14.44)	4584 (13.41)	
Current		1,008,246 (26.46)	4177 (18.62)	7460 (21.82)	
Drinking	*n*, (%)				<0.0001
None		1,948,697 (51.14)	13,117 (58.48)	18,910 (55.31)	
Mild		1,554,838 (40.8)	8006 (35.69)	13,081 (38.26)	
Heavy		306,995 (8.06)	1307 (5.83)	2196 (6.42)	
Regular exercise	*n*, (%)	679,684 (17.84)	4155 (18.52)	6020 (17.61)	0.0148
BMI, kg/m^2^ (group)	*n*, (%)				<0.0001
<18.5		140,957 (3.7)	933 (4.16)	1509 (4.41)	
18.5–23		1,485,817 (38.99)	8924 (39.79)	14,097 (41.23)	
23–25		937,790 (24.61)	5269 (23.49)	8067 (23.6)	
25–30		1,110,011 (29.13)	6499 (28.97)	9408 (27.52)	
≥30		135,955 (3.57)	805 (3.59)	1106 (3.24)	
BMI, kg/m^2^		23.71 ± 3.47	23.62 ± 3.28	23.49 ± 3.25	<0.0001
Waist circumference, cm		80.24 ± 9.51	79.87 ± 9.64	79.39 ± 9.53	<0.0001
Diabetes mellitus	*n*, (%)	330,158 (8.66)	2525 (11.26)	2757 (8.06)	<0.0001
Hypertension	*n*, (%)	1,022,277 (26.83)	7132 (31.8)	9022 (26.39)	<0.0001
Dyslipidemia	*n*, (%)	691,809 (18.16)	4827 (21.52)	6752 (19.75)	<0.0001
CKD	*n*, (%)	260,196 (6.83)	1996 (8.9)	2517 (7.36)	<0.0001
SBP, mmHg		122.45 ± 15.05	121.63 ± 15.12	121.25 ± 14.87	<0.0001
DBP, mmHg		76.33 ± 10.07	75.62 ± 10	75.47 ± 9.91	<0.0001
Fasting glucose, mg/dL		97.29 ± 23.93	97.45 ± 24.86	95.59 ± 21.84	<0.0001
Total cholesterol, mg/dL		195.37 ± 41.52	193.75 ± 44.78	195.33 ± 41.43	<0.0001
HDL cholesterol, mg/dL		56.45 ± 32.77	57.46 ± 37.24	57.74 ± 33.53	<0.0001
LDL cholesterol, mg/dL		121.34 ± 217.11	120.54 ± 208.62	121.15 ± 211.59	0.8456
TG, mg/dL		112.86 (112.79–112.92)	109.56 (108.73–110.4)	109.52 (108.85–110.2)	<0.0001
Treatment					
Topical agents	*n*, (%)	-	19,873 (88.6)	25,626 (74.96)	<0.0001
Systemic agents	*n*, (%)				
Steroid		-	-	33,988 (99.42)	<0.0001
Azathioprine		-	-	28 (0.08)	<0.0001
Cyclosporine		-	-	430 (1.26)	<0.0001
Methotrexate		-	-	64 (0.19)	<0.0001
Mofetil		-	-	19 (0.06)	<0.0001

Continuous variables are presented as the mean ± SD and categorical variables as number (percentage). BMI, body mass index; SBP, systolic blood pressure; DBP, diastolic blood pressure; TG, triglycerides; HDL, high-density lipoprotein; LDL, low-density lipoprotein; CKD, chronic kidney disease.

**Table 2:** Incidence rate and risk of malignancy according to AD severity.

	AD Severity	N	Event	Duration ^a^	Rate ^b^	Model 1	*p*-Value	Model 2	*p*-Value	Model 3	*p*-Value
Cancer	No AD	3,810,530	201,458	30,708,388.67	6.56036	1 (Ref.)	<0.0001	1 (Ref.)	0.0052	1 (Ref.)	0.0036
	Mild	22,430	1384	179,514.94	7.70966	1.175 (1.115, 1.239)		1.061 (1.007, 1.119)		1.061 (1.006, 1.118)	
	Moderate to severe	34,187	1899	274,374.31	6.9212	1.055 (1.008, 1.104)		1.057 (1.01, 1.106)		1.061 (1.014, 1.11)	
Renal cancer	No AD	3,810,530	5157	31,355,301.55	0.16447	1 (Ref.)	0.004	1 (Ref.)	0.0015	1 (Ref.)	0.0017
	Mild	22,430	30	184,193.3	0.16287	0.99 (0.692, 1.418)		0.92 (0.643, 1.318)		0.897 (0.626, 1.284)	
	Moderate to severe	34,187	69	280,439.45	0.24604	1.496 (1.18, 1.897)		1.54 (1.215, 1.953)		1.533 (1.209, 1.944)	

Values are expressed as HRs (95% CIs) calculated in a Cox proportional hazard regression analysis. MODEL 1: Unadjusted. MODEL 2: Adjusted for baseline age and sex. MODEL 3: Adjusted for baseline age, sex, hypertension, DM, dyslipidemia, smoking, drinking, exercise, and BMI. ^a^ Duration is in person-years. ^b^ Per 1000 person-years.

**Table 3:** Incidence rate and risk of renal malignancy in the study participants by AD treatment modality.

Treatment	N	Renal Cancer	Duration ^a^	Rate ^b^	HR (95% CI)					
					Model 1	*p*-Value	Model 2	*p*-Value	Model 3	*p*-Value
No AD	3,810,530	5157	31,355,301.55	0.16447	1 (Ref.)	0.0035	1 (Ref.)	0.0028	1 (Ref.)	0.0034
Only topical agents	19,892	24	163,440.45	0.14684	0.893 (0.598, 1.334)		0.847 (0.567, 1.265)		0.828 (0.555, 1.237)	
Systemic—Steroid	33,988	68	278,813.78	0.24389	1.483 (1.168, 1.884)		1.527 (1.202, 1.939)		1.519 (1.196, 1.93)	
Systemic—Others	2737	7	22,378.52	0.3128	1.903 (0.907, 3.993)		1.554 (0.741, 3.261)		1.476 (0.703, 3.098)	

Values are expressed as HRs (95% CIs) calculated in a Cox proportional hazard regression analysis. MODEL 1: Unadjusted. MODEL 2: Baseline age and sex. MODEL 3: Baseline age, sex, hypertension, DM, dyslipidemia, smoking, drinking, exercise, and BMI. ^a^ Duration is in person-years. ^b^ per 1000 person-years.

## Data Availability

Data available upon request from the authors.
